# Alcohol-Related Cognitive Impairments: An Overview of How Alcoholism May Affect the Workings of the Brain

**Published:** 1995

**Authors:** Denise L. Evert, Marlene Oscar-Berman

**Affiliations:** Denise L. Evert, Ph.D., is a National Institutes of Health postdoctoral trainee in the Department of Neurology at Boston University School of Medicine and the Department of Veterans Affairs Medical Center, Boston, Massachusetts. Marlene Oscar-Berman, Ph.D., is a professor in the departments of psychiatry and neurology, Boston University School of Medicine, and a research scientist in the Psychology Research Service, Department of Veterans Affairs Medical Center, Boston, Massachusetts

**Keywords:** cognitive process, AOD impairment, AOD dependence, brain, AOD abstinence, Korsakoff’s syndrome, scientific model

## Abstract

People with alcoholism express cognitive deficits of varying type and severity. Theoretical models of impaired cognition are intended to explain the deficit patterns by relating them to the brain structures or brain processes that may be damaged. Although no model has thus far succeeded in defining adequately all the impairments alcoholics experience, process-oriented models are proving useful as tools for describing alcohol-related cognitive deficits.

What does it mean to have cognitive impairments? Basically, it means to experience problems with what one knows and how one comes to know it. More formally, the term “cognition” has been defined as “mental activities [that] involve the acquisition, storage, retrieval, and use of knowledge” (Matlin 1989, p. 2). Thus, cognition encompasses a wide variety of mental processes, such as using one’s eyes and ears to perceive the outside world, forming memories and mental images, using and understanding language, and engaging in reasoning, problem-solving, and decision-making. In essence, cognition can include practically any mental process that occurs between the initial intake of stimulus energy by one’s senses and the execution of motor responses with one’s muscles.

Researchers have long linked cognitive-functioning impairments with alcoholism.^1^ This article describes cognitive deficits in long-term abstaining alcoholics (i.e., alcoholics who have abstained from consuming alcohol for a minimum of 1 month)^2^ and reviews different theoretical models proposed to explain these deficits. First, a brief description is provided of an uncommon but severe consequence of alcoholism on cognitive functioning, referred to as “alcoholic Korsakoff’s syndrome.” Next, the article reviews a growing body of evidence demonstrating cognitive impairments in alcoholics with no obvious signs of Korsakoff’s syndrome. Finally, a variety of models used to characterize the nature of these cognitive deficits is presented.

## Cognitive Functioning Deficits in Abstinent Alcoholics

Alcoholic Korsakoff’s syndrome is characterized most notably by cognitive impairments in memory (i.e., anterograde amnesia—an inability to remember new information for more than a few seconds) as well as deficits in abstraction and problem-solving (Jacobson et al. 1990). Despite these cognitive-functioning impairments, overall intelligence, as measured by IQ tests, usually remains intact. This is because most memories formed before the onset of prolonged heavy drinking remain preserved, whereas memories recently acquired are not preserved. Thus, general intelligence is spared, because the types of information and the abilities tapped by IQ tests often have not been acquired recently and can be retrieved from memory.^3^

Some researchers have found evidence of an enzyme deficiency in alcoholic Korsakoff patients that prevents their bodies from metabolizing thiamine (vitamin B) efficiently (Blass and Gibson 1977; Bowden 1990; see also the article by Langlais, pp. 113–121). This deficiency could be genetically inherited or environmentally induced. Therefore, people who suffer from a metabolic disorder that does not permit the body’s normal use of thiamine or who do not eat enough thiamine-containing foods (e.g., if alcohol comprises most of their diets) may be at risk for developing cognitive impairments (and structural brain changes, which may occur before the impairments arise) associated with Korsakoff’s syndrome.^4^ Regardless of the actual cause of alcoholic Korsakoff’s syndrome, its incidence is quite low. According to one estimate, only 10 per 1 million (.001 percent) of patients admitted for the first time into a psychiatric clinic exhibit characteristics of alcoholic Korsakoff’s syndrome (Centerwall and Criqui 1978).

Alcoholics who do not develop Korsakoff’s syndrome still may show signs of cognitive impairment. In fact, the once commonly held view that those alcoholics without evidence of Korsakoff’s syndrome were cognitively “intact” (see Lishman 1990, p. 635) has been abandoned in light of accumulating evidence indicating that cognitive impairments (and changes in brain structure) can exist in alcoholics who do not exhibit obvious clinical signs of anterograde amnesia (i.e., the symptom that indicates that a person has Korsakoff’s syndrome). Researchers have gathered this evidence by developing refined and sensitive tests of psychological functioning. The tests are given to alcoholics and to normal control subjects who are matched for important characteristics, such as age, gender, education, and ethnic background, and their scores are compared. When statistical analyses reveal significant differences in performance between alcoholics and control groups on the tests, researchers conclude that the cognitive impairments are related to alcoholism.

**Figure 1:**
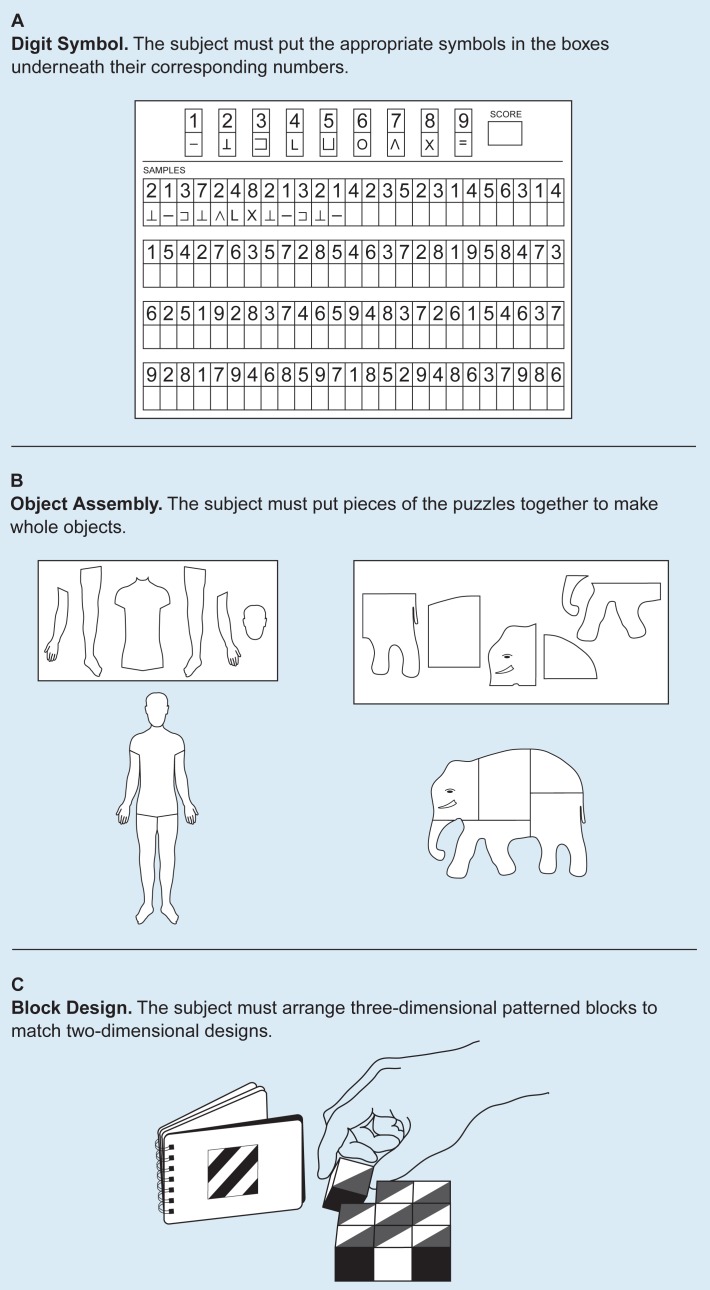
Examples from a commonly used IQ test (WAIS–R) assessing visuospatial abilities (the ability to deal with objects in three-dimensional space). All three subtests are timed, and subjects are asked to work as quickly as they can.

Within the past 25 years, cognitive deficits found to be associated with alcoholism have included slowed processing of information, difficulty in learning new material, deficits in abstraction and problem-solving, and reduced visuospatial abilities (i.e., the capacity to deal with objects in two-dimensional or three-dimensional space; Ellis and Oscar-Berman 1989). Reduced visuospatial abilities have been reported most consistently. Sections of IQ tests (called performance subscales) that are especially sensitive to visuospatial abilities commonly are used to assess deficits in this area. These IQ subscales usually impose time limits and include such tasks as substituting symbols for numbers, assembling small jigsaw puzzles, or arranging colored cubes in the same pattern as that presented in a picture (figure 1).

### The Continuum of Cognitive Change

Scientists have proposed that cognitive changes in alcoholics develop progressively (and are correlated with the duration and degree of a person’s alcohol use), and, thus, impairments in cognitive functioning can be represented along a continuum (Ryback 1971). The continuum encompasses, at one end, abstainers and social drinkers who exhibit no signs of cognitive impairment and, at the other end, alcoholics with Korsakoff’s syndrome who exhibit severe deficits in memory and other cognitive functions. Chronic alcoholics who do not suffer from Korsakoff’s syndrome but who exhibit signs of mild to moderate cognitive impairment are placed along this continuum between the two extremes. This has come to be referred to as the “continuum hypothesis” and suggests that chronic alcoholics should display at least some of the same cognitive changes present in people with Korsakoff’s syndrome (Ryan and Butters 1980).

Studies testing the claim set forth by the continuum hypothesis have shown mixed results. For example, Ryan and Butters (1980) demonstrated that when required to perform demanding learning and memory tests, different subjects’ patterns of performance fell along a continuum: Alcoholics with Korsakoff’s syndrome performed more poorly than did alcoholics without Korsakoff’s syndrome who spontaneously had reported memory problems (but showed no clinical signs of amnesia). These people, in turn, performed less well than did alcoholics with no complaints of memory problems, who, in turn, performed less well than did nonalcoholics in a control group. In addition, the researchers provided evidence that alcoholics who had not complained of memory problems still exhibited memory deficits when the tasks were made more difficult, thereby increasing information-processing demands.

Conversely, other investigators (Cermak et al. 1974) have demonstrated that the pattern of performance that alcoholics demonstrate on memory tests is similar to that of normal control subjects, and their performance, in turn, is qualitatively different from those alcoholics with Korsakoff’s syndrome. More recently, Delin and Lee (1992) reviewed evidence indicating that social drinkers, who are presumed to occupy a place on the continuum between that of abstainers and alcoholics, do not show the deleterious effects of alcohol on mental functioning. Thus, the evidence in this review regarding cognitive changes does not support the continuum hypothesis.

The implicit claim of the continuum hypothesis is that cognitive-functioning impairments should correlate with the extent of alcohol consumption. Assuming that alcohol-related structural brain changes account for cognitive decline, it would be expected that specific measures of prior alcohol consumption—such as quantity of drinks consumed per day, frequency (i.e., number of drinking days per week), and duration (i.e., number of years) of drinking—would correlate with test performance among alcoholics. Researchers who study alcohol-related cognitive changes have not reported this consistently. However, when a significant correlation is found between cognitive functioning in alcoholics and some measure of alcohol consumption, it is usually in the expected direction—that the greater the consumption of alcohol, the worse the performance on cognitive tasks.

### Variables Affecting Cognitive Deficits

Widespread individual differences exist in the manifestation of cognitive deficits in abstinent alcoholics, with 50 to 85 percent of those alcoholics without Korsakoff’s syndrome exhibiting signs of cognitive decline (see Parsons 1993). Thus, anywhere from 15 to 50 percent of abstinent alcoholics may not exhibit any obvious signs of cognitive impairment. Although no single reason can be given to explain the inconsistent relationship between alcohol consumption measures and test performance, researchers have explored numerous possible variables that may account for the relationship (for a review, see Parsons 1993), including the following subject characteristics:

- Age
- Gender
- Diet
- Associated medical problems (e.g., liver disease)
- Emotional difficulties
- The subject’s general motivational level (e.g., a subject’s level of cooperation, effort, or desire to do well may affect performance on a cognitive test)
- Cognitive deficits that are related to childhood behavior problems (e.g., conduct disorder, attention deficit disorder, learning disabilities, or hyperactivity), antisocial personality before the onset of alcoholism, or family history of alcoholism (i.e., premorbid disorders).

One approach to explaining the inconsistency in test results has been to test different subgroups of alcoholics that are defined based on one or more of these variables. Researchers look for differences in the degree of cognitive impairment manifested (as indicated by the test scores) by each of the various groups. However, conclusive evidence has yet to be found to support the possibility that any one of these variables alone could completely and consistently account for alcoholics’ cognitive impairments (Parsons 1993). Thus, the most plausible hypothesis is that cognitive deficits in alcoholics result from prolonged alcohol ingestion, which impairs the way the brain normally works (i.e., the functional brain states) in certain vulnerable alcoholics. Characterizing what makes certain abstinent alcoholics vulnerable remains open for debate.

## Looking at Brain Structure

One way of viewing cognitive changes in abstinent alcoholics is to emphasize alcohol-related changes in brain structure that may cause the impairments. The brain encompasses a layer of tissue that lies just underneath the skull. This layer, called the cerebral cortex, is thought to “house” many cognitive functions (for locations and definitions of this and other brain areas, see the figure, pp. 136–137). According to one view (Lishman 1990), shrinkage of the cerebral cortex, as well as possible atrophy of basal forebrain regions, is thought to be caused by alcohol’s direct neurotoxic effects. Furthermore, thiamine deficiency may result in damage to a region deep within the brain called the diencephalon (perhaps because blood vessels break in that region when the body’s thiamine levels are deficient).

According to this view, alcoholics who are susceptible to alcohol toxicity but not to thiamine deficiency may develop permanent or transient cognitive deficits associated with cortical shrinkage. Those alcoholics who are susceptible to thiamine deficiency alone will develop a mild or transient Korsakoff state with anterograde amnesia as a salient feature. Alcoholics with dual vulnerability, suffering from a combination of alcohol neurotoxicity and thiamine deficiency, will experience widespread damage to large regions of the cerebral cortex as well as to structures deep within the brain. Consequently, these people will exhibit severe anterograde amnesia and other cognitive impairments. The following discussion of theoretical models that have been proposed to explain cognitive deficits primarily will consider those people mentioned in the first group—those with alcohol problems whose cognitive impairments most likely are related to cortical brain changes and who exhibit no clinical signs of anterograde amnesia.

## Theoretical Models of Cognitive Impairment

Theoretical models used to explain cognitive functioning can be categorized based on the fundamental approach that the researchers using them adopt. Some theories apply knowledge about the brain’s structure to explain a decline in mental functioning. The theories using this approach, called a structure-function relationship, compare the performance of patients with known damage, either localized to specific regions of the brain or diffusely distributed, with the performance of people with brain damage of unknown or uncertain location (which may or may not be a result of alcohol abuse). Theories based on another approach, referred to here as a “process-oriented” approach, examine the underlying nature of the observed cognitive functional decline with little or no direct reference to brain structure. The process-oriented approach uses models that psychologists have constructed to describe memory and other mental processes.

Both approaches—the structure-function relationship and the process-oriented approach—have been used to help explain cognitive-functioning impairments in abstinent alcoholics. The hypotheses based on these approaches are not necessarily mutually exclusive, however. No single hypothesis can yet explain all the research findings of cognitive impairments in alcoholics. Instead, these hypotheses apply to particular aspects of the empirical data discussed here. Thus, each model provides only a partial answer to how alcoholism impairs cognitive function.

### Interplay Between Brain Structure and Function

Based on the performance of patients with specific regions of brain damage unrelated to alcoholism (for example, damage from strokes, trauma, tumors, or other disease), researchers have gained a clearer understanding of which areas of the brain are important for different aspects of cognitive functioning. The cognitive deficits manifested in alcoholics correspond to those seen in patients whose brain damage is unrelated to alcoholism (see Parsons 1993). Researchers most often have connected the following regions of the brain with alcohol-related cognitive impairments and have developed models based on them: the right half or hemisphere (the right hemisphere model), cortical tissue diffusely distributed throughout both the left and right hemispheres (the diffuse brain dysfunction model), and the frontal lobe systems (the frontal lobe model).

#### The Premature Aging Hypothesis

Scientists have obtained independent evidence in older nonalcoholics (over age 50) supporting two of the structure-function models (i.e., the right hemisphere model and the diffuse brain dysfunction model; both are discussed below). These findings of similar cognitive profiles (and changes in brain structure) found both in alcoholics and older nonalcoholics suggest that alcoholism may accelerate normal aging or cause premature aging of the brain. The following sections first explain the premature aging hypothesis, then describe the three structure-function models, examining how they attempt to explain cognitive deficits in alcoholics. Each model also is applied to the premature aging hypothesis to determine whether it supports or opposes the hypothesis.

By the 1960’s enough circumstantial evidence had accumulated to lead some researchers to propose that chronic alcoholism is associated with premature aging of the brain (for reviews, see Freund and Butters 1982 and Wood and Elias 1982). This claim, referred to as the “premature aging hypothesis,” originally evolved from observations concerning structural brain changes. For example, Wilkinson and Carlen (1982) described a study in which the brain scans of alcoholics were compared with those of a group of patients who had a variety of neurological conditions unrelated to alcoholism. The participants’ ages spanned five age groups, from the twenties through the sixties. The researchers found that the brains of alcoholics, as well as those of older nonalcoholics, appeared to be shrunken inside their skulls. Decades earlier, Courville (1966) described this same feature of alcoholics’ brains, and he likened it to the brain shrinkage associated with normal chronological aging. More recently, Pfefferbaum and colleagues (1992) used magnetic resonance imaging (MRI) techniques and found evidence of increased brain tissue loss in alcoholics, compared with nonalcoholics, even after their ages had been taken into account. Considered together, these findings provide evidence that alcoholics and aging nonalcoholics show atrophy of the cerebral cortex. As discussed below, however, studies revealing different patterns of cognitive impairment as a result of this brain atrophy have not always supported the premature aging hypothesis.

Scientists have proposed two versions of the premature aging hypothesis: the accelerated aging version, which proposes that aging may be accelerated by alcoholism at whatever age alcohol abuse begins, and the increased vulnerability version, which proposes that vulnerability to alcohol-related brain damage is magnified in alcohol-abusing people over age 50 after the normal manifestations of aging begin (Ellis and Oscar-Berman 1989). Thus, according to the accelerated aging version, young alcoholics may become old before their time. And according to the increased vulnerability version, older people who abuse alcohol may suffer proportionately more age-related cognitive changes than their non-alcoholic peers because of their aged brains’ increased vulnerability to alcohol-related damage. To date, the controversy about which version of the premature aging hypothesis is best supported by research is not yet resolved.

Although the above predictions are stated in terms of cognitive changes, the same predictions may apply to changes in brain structure. For example, in addition to the MRI findings mentioned earlier, Pfefferbaum and colleagues (1992) found that older alcoholics exhibited more tissue loss than younger alcoholics, which suggests that older alcoholics may be particularly susceptible to alcohol’s effects. These findings support the increased vulnerability version of the premature aging hypothesis.

#### Right Hemisphere Model

In most people, the brain’s functions are organized asymmetrically: The left hemisphere is dominant for language-related functions, such as speaking and understanding spoken words, whereas the right hemisphere is dominant for nonverbal skills, such as reading maps, solving jigsaw puzzles, listening to music, or performing motor skills. This is called normal asymmetrical brain function, because each hemisphere of the brain appears to play a more important role in a particular aspect of cognitive functioning than does the other hemisphere.

Researchers have hypothesized that right-brain functions are more vulnerable to alcoholism’s effects than are left-brain functions (for a review, see Ellis and Oscar-Berman 1989). They have based this right hemisphere model on findings that alcoholics generally show a steeper decline on nonverbal tasks than on verbal tasks on most IQ tests (figure 1). For example, on a common IQ test, the Wechsler Adult Intelligence Scale–Revised (WAIS–R; Wechsler 1981), alcoholics often perform abnormally poorly on the digit symbol, object assembly, and block design subtests, all of which assess nonverbal information processing (Ellis 1990; see also the article on cognitive assessment by Nixon, pp. 97–103). Similar deficits appear when both nonverbal and verbal materials are used in tasks that assess mental functions such as memory and attention. These types of cognitive deficits reported in long-term chronic alcoholics resemble the deficits observed in patients with damage to the right hemisphere of the brain that is unrelated to alcoholism.

In studies applying the right hemisphere model to premature aging, older nonalcoholics have exhibited performance patterns similar to alcohol-induced deficits usually associated with the decline in right hemisphere functioning. This finding supports the view that alcoholism accelerates normal aging and/or results in increased vulnerability to brain damage.

In examining the right hemisphere model, Ellis (1990) found that older alcoholics performed significantly worse on the performance subtests of the WAIS–R compared with younger alcoholics and with both younger and older nonalcoholic control subjects, thus providing support for accelerated aging in chronic alcoholics.

Although the right hemisphere model is supported by research as it relates to alcoholism and to normal aging separately, several weaknesses of this model should be noted (for review, see Ellis and Oscar-Berman 1989). First, the findings by Ellis (1990) mentioned above have not been reproduced, and the majority of the evidence does not strongly support either version of the premature aging hypothesis in relation to this model. Second, this model is descriptive rather than explanatory. That is, no explanation is provided for why the right hemisphere of the brain per se would be differentially sensitive to the effects of alcoholism and aging. A third problem with this model is that alcoholics have been shown to manifest verbal cognitive deficits (which are associated with the left brain) in addition to nonverbal patterns of deficits when sensitive tasks are used. Thus, when differences are found between alcoholics’ performance on verbal and nonverbal tasks, it may be explained simply by the specific demands of the tasks rather than by solely right brain deficits.

For example, both alcoholic subjects and elderly control subjects, despite differences in cognitive performance patterns (mentioned earlier), exhibit a performance pattern on laterality tasks (see box, p. 94) that is similar to that of nonalcoholic control subjects. Thus, they do not exhibit deficits on these tasks (Ellis 1990). Therefore, these laterality findings do not support the right hemisphere model. These findings suggest that the functional deterioration associated with alcoholism and aging influence both hemispheres of the brain. This view is discussed below in more detail.

**Brain Laterality Tasks**

Scientists have suggested that a more sensitive method than verbal and nonverbal tasks for examining differences in asymmetrical brain functioning is to employ dichotomous stimulation techniques (Ellis and Oscar-Berman 1989). These techniques, also known as brain laterality tests, challenge the two cerebral hemispheres by presenting information simultaneously to both halves of the brain, either through the visual, auditory, or tactile sensory modalities. The brain is organized so that information entering the right side of the body through the right ear, hand, or visual field goes first to the left side of the brain before crossing over to the right side; the pattern is reversed for information entering the left side of the body. Using audition as an example, normal control subjects (with neither brain damage nor alcoholism) have a clear advantage in processing musical sounds that come first into the right side of the brain from the left ear. Alternatively, normal control subjects have a clear advantage in processing words when information is presented initially to the brain’s left hemisphere through the right ear. For further discussion of the asymmetries of brain function in alcoholism, see Oscar-Berman 1992.

#### Diffuse Brain Dysfunction Model

According to this view, researchers propose that chronic alcoholism results in global cognitive dysfunction, with subjects exhibiting a wide variety of cognitive deficit patterns. These cognitive changes suggest mild diffuse brain dysfunction. For example, Parsons and colleagues (1990) administered a battery of verbal and visuospatial tests comprising different factors that employed both left and right hemisphere functioning. The researchers found that the alcoholics performed significantly poorer than the nonalcoholic controls on tasks comprising all factors tested, suggesting that both left and right hemisphere functions were compromised.^5^ This finding lent support to the diffuse brain dysfunction model.

Looking more closely at this model, deficits in some cognitive functions may result from task difficulty alone and/or decline in the brain’s overall cognitive capacity, as opposed to specific deficits related to lesions in a certain brain region. Because of its lack of specificity, the diffuse brain dysfunction model more adequately explains these findings of left and right hemisphere deficits than do the other models. This model can explain adequately a large portion of the deficit patterns observed in alcoholics.

Because deficit patterns in chronic alcoholics have been shown to mimic some cognitive changes in older nonalcoholics, researchers have proposed that the model also supports the accelerated aging hypothesis. However, evidence supporting the diffuse brain dysfunction model does not consistently meet the specific predictions of the premature aging hypothesis. The model’s primary weakness is that it cannot be disproved easily because it does not make differential predictions about the performance of alcoholics and normal control subjects on different kinds of cognitive-functioning tests. In comparison with the right hemisphere model, for example, the model suggesting diffuse brain damage does not link specific impairments with distinct regions of brain damage.

#### Frontal Lobe System Dysfunction Model

The last model based on examining the relationship between brain structure and cognitive function suggests that alcoholism selectively disrupts those cognitive functions normally ascribed to the frontal lobes of the brain and their connections with other brain regions (called frontal lobe systems or frontal systems). Along with evidence of changes in frontal system brain structure, this model is based on findings that alcoholics show personality changes and cognitive impairments similar to those of patients with frontal system brain damage unrelated to alcoholism (for a review, see Oscar-Berman and Hutner 1993).

Alcoholics and patients with frontal lobe system damage resulting from causes other than alcohol have been shown to exhibit impaired impulse control, lack of insight, and difficulty adapting to change. In addition, they perform poorly on tests of planning, organizing, problem-solving, and abstraction. Probably the most consistent finding in alcoholics that implicates deficits in frontal lobe functioning is abnormal perseverative responding (i.e., repetition of a previous behavior or response pattern despite feedback indicating that such responses are no longer correct or appropriate).

Researchers also have found supporting evidence for the frontal system model from studies in which alcoholics and nonalcoholics were asked to perform tests known to reveal deficits caused by lesions in frontal brain systems in animals (Oscar-Berman et al. 1992). One such task used with nonhuman primates with frontal brain lesions is the delayed-response (DR) task in which a reward is placed into a hole under one of two identical flat wooden covers that differ only in their location on a tray. In this task the subject must notice and remember where the experimenter placed the reward in each session. As soon as the holes are covered with the boards, a screen is lowered between the experimenter and the subject. After a short delay (usually between 0 and 60 seconds), the experimenter raises the screen so that the tray containing the reward is within the subject’s reach, and the subject must choose which board covers the reward. Another task, referred to as delayed alternation (DA), is similar except that the subject must now learn to alternate its responses from left to right. In this task, the subject must learn to inhibit previously rewarded responses so that it can make the correct choice on subsequent trials (e.g., it must be able to remember that the reward is hidden on the right, even if it had been hidden on the left in the previous trial).

Two large subdivisions of prefrontal cortex are considered important in normal DR and DA performance. In both the human and nonhuman primate literature, evidence suggests that the functions related to successful DR performance rely more heavily on the integrity of the dorsolateral prefrontal system of the brain (located close to the skull near the temples), whereas those functions related to successful DA performance rely more heavily on the integrity of the orbitofrontal system of the brain (located behind and above the eyes).

One study used a task assessing DR performance in both the visual and auditory sensory modalities in humans (Oscar-Berman et al. 1992). The study found that alcoholic Korsakoff patients showed clear deficits on this task compared with the non-Korsakoff alcoholics and normal control subjects (especially when demands were placed on visual processing time and on short-term memory). Based on these observations, the researchers hypothesized that prefrontal cortical structural change, particularly involving the orbitofrontal system, must be a prominent characteristic of alcoholic Korsakoff’s syndrome. The non-Korsakoff alcoholics showed little evidence of damage to frontal systems by these tests, either because the tests were not sufficiently sensitive to mild deficits or because frontal damage is minimal or absent in alcoholism uncomplicated by Korsakoff’s syndrome.

When applying this model to the premature aging hypothesis, no similar patterns of cognitive change related to possible frontal system dysfunction have been found in older nonalcoholic populations. This suggests that the premature aging hypothesis may not be adequate to explain the pattern of performance deficits displayed in chronic alcoholics with respect to frontal lobe system dysfunction.

### The Process-Oriented Approach

An alternative way of viewing the effects of alcoholism on cognitive functioning from the structure-function relationship is to examine and define the underlying cognitive processes that are impaired in chronic alcoholics. In contrast to the previously described models, which focus on ultimate performance measures, the process-oriented approach emphasizes the underlying cognitive mechanisms involved, sometimes with little or no reference to brain structure.

Within the domain of memory functioning, researchers have used this approach by defining different dimensions for processing memory. These dimensions of memory have specific terms associated with them (e.g., short-term vs. long-term memory, declarative vs. procedural memory, and episodic vs. semantic memory). For example, declarative memory refers to knowing a particular piece of information (e.g., a phone number), and procedural memory refers to being able to perform a particular task without necessarily knowing when or where one learned how to do it (e.g., tying shoe-laces). Similarly, episodic memory refers to memory for specific events, facts, or episodes (e.g., it snowed yesterday), whereas semantic (or knowledge) memory is general, organized knowledge about the world (e.g., 52 weeks compose a year). An individual may be impaired in one dimension of processing but not in the other. Each dimension has been used to help researchers understand preserved and impaired memory abilities in a variety of subject populations, including alcoholic Korsakoff patients (for a review, see Cermak 1990).

The process-oriented approach also has been used to examine a broad range of cognitive functions in non-Korsakoff alcoholics. Nixon and Parsons proposed a model called a component-process model (Parsons and Nixon 1993; Nixon 1993). Their model encompasses two information stores, referred to as the “episodic store” and the “knowledge information store,” within which three component processes (described below)—availability, access, and efficiency—may operate. The approach’s goal is to determine which processes and/or stores are impaired in alcoholics to cause cognitive deficits. Learning and memory of context-bound information (e.g., recalling where you were or what you were doing when you heard of the assassination of President Kennedy or of the explosion of the Challenger spacecraft) is housed in the episodic store. Processes related to the use of language, logic, and semantic knowledge (e.g., knowing that President Kennedy was assassinated), in addition to processes related to abstracting and problem-solving, are housed within the knowledge-information store.

Within each store, the three component processes may operate: “availability” refers to the persistence of information over time, “access” refers to the ability to retrieve previously acquired information, and “efficiency” refers to the ability to use accurate or relevant information while ignoring or disregarding inaccurate or irrelevant information. According to the component-process model, the nature of the deficit differs for each of these three processes. Availability deficits are characterized by impairments in accuracy as measured by increased errors. Access impairments are characterized by a slowing of behavior that can be measured by increases in a person’s response time. And efficiency deficits are characterized by a person’s inability to ignore irrelevant or inaccurate information.

Thus far, research suggests that efficiency processes within the knowledge-information store may be particularly susceptible to alcoholism. For example, Nixon and Parsons (1991) used the “plant task” to determine whether alcoholics and controls exhibit differences in abstraction ability, which, as noted above, is housed within the knowledge-information store. This test has more ecological validity than other common tests of abstraction—its relevance to real-world functioning is more apparent than most laboratory tasks used to assess different aspects of cognitive functioning.

For this task, subjects are shown four plants, two of which are healthy and two of which are unhealthy. After hearing the individual treatments each plant has received (e.g., type of plant food and amount of water), participants are asked to decide the health of a plant they cannot see based on a description of its treatment (for further discussion, see the article by Nixon, pp. 97–103). In addition, the subjects are asked to describe how they know that the plant is healthy or unhealthy; in other words, they must identify the relevant variable related to the plant’s outcome.

The study by Nixon and Parsons (1991) predicted that availability deficits would be characterized by a subject’s inability to identify the relevant variable in the task and that efficiency deficits would be characterized by the inclusion of irrelevant variables in describing the reason for the plant’s outcome. The results indicated that although no group differences existed in the ability to predict correctly the fate of the unseen plant, the alcoholics experienced significantly greater difficulty than the control subjects in their ability to isolate the relevant variable, having more problems with ignoring irrelevant information in their explanations of the plant’s outcome. The researchers concluded that this deficit was indicative of efficiency impairments within the knowledge-information store. Although it is premature to link deficits in efficiency with any particular brain system, it is hoped that research will bring these two areas of investigation together.

## Summary and Conclusions

Much of the evidence reviewed here supports the idea that brain damage associated with long-term alcoholism can be extensive and that a wide range of variability in both the severity and types of cognitive impairments exists. The evidence reviewed focuses on the specific impairments in cognitive functioning related to chronic alcoholism. The hypotheses discussed attempt to specify behavioral changes in relation to brain structure and function. This strategy is intended to help link patients’ alcohol-related cognitive impairments to brain-damaged systems. If research can make such a connection, treatment strategies can be planned that draw on patients’ cognitive functions controlled by undamaged brain systems.

Differences observed between right and left hemisphere functional decline in alcoholics appear to be related to differential task demands rather than to a specific disruption of one type of cognitive ability. Evidence from brain imaging studies (not reviewed here), as well as postmortem examination of alcoholics’ brains, supports the view that the damage is diffuse and involves many cortical and subcortical regions. Although evidence indicates that non-Korsakoff alcoholics have somewhat more vulnerability of the frontal lobes than other brain regions to structural damage, there is no substantial evidence that alcoholics have cognitive impairments similar to nonalcoholic patients with frontal lobe damage. The evidence to date for the frontal system model of cognitive impairment in non-Korsakoff alcoholics is at best mixed and probably indistinguishable from the diffuse brain dysfunction model. Finally, the idea that alcoholism is associated with premature aging is not strongly supported in the literature.

The process-oriented approach presented here describes an alternative way of assessing the nature of the cognitive deficits in abstinent alcoholics. This approach takes a closer look at the specific processes underlying different aspects of cognitive functioning rather than just assessing ultimate performance. Although the specific component-process model presented here is fairly new and awaits further testing, the results generated thus far suggest that it will be a useful tool in helping to describe alcohol-related cognitive impairments.

As noted earlier, the two main approaches described here—the structure-function relationship and the process-oriented approach—are not necessarily mutually exclusive, and a combination of both would be beneficial in pursuing this line of research. Future research hopefully will generate a single, comprehensive model of alcohol-related cognitive impairment. For example, scientists might be able to suggest possible brain systems involved with the specific underlying functional mechanisms that are particularly susceptible to chronic alcoholism.

## Footnotes

1 The term “alcoholism” in this article refers to the criteria for alcohol dependence as defined in the American Psychiatric Association’s *Diagnostic and Statistical Manual of Mental Disorders, Fourth Edition* (DSM–IV). The term “alcohol abuse” also refers to the definition given in DSM–IV.

2 Scientists study cognitive deficits among abstinent alcoholics because they are interested in describing changes in cognitive function that result from changes in brain structure. In alcoholics who are not abstinent, it is difficult to separate the cognitive changes that result from acute exposure to alcohol from the cognitive changes that result from long-term structural alterations in their brains.

3 Although Korsakoff’s syndrome is usually preceded by a more acute stage of neurological problems, which includes confusion, consciousness impairments, difficulty moving eye muscles, and problems with gross muscle control (collectively referred to as “Wernicke’s encephalopathy”), the present discussion specifically addresses the long-term residual effects of alcoholism on cognitive functioning in abstinent alcoholics.

4 People who have a metabolic deficiency that impairs their ability to absorb thiamine may develop Korsakoff’s syndrome without consuming alcohol at all. However, this article refers to those people for whom the deficiency acts in concert with alcoholism to produce alcoholic Korsakoff’s syndrome.

5 This finding is at variance with the results of laterality tests that are reported here. Scientists have yet to determine why this discrepancy exists.
